# 

**Published:** 1902-03

**Authors:** 


### BOOKS RECEIVED.

A Manual of Ophthalmoscopy. For Students and General Practitioners. By J. E. Jennings, M. D. (Univ. of Penna.), Author of Color-Vision and Color-Blindness, etc.; formerly Clinical Assistant, Royal London Ophthalmic Hospital. Duodecimo, pp. 180, with 95 illustrations and 1 colored plate. Philadelphia: P. Blakiston's Son & Co 1902. [Price, $1.25 net.]

The Practical Medical Series of Year Books. Ten volumes. Issued monthly, under the general editorial charge of Gustavus P. Head, M. D., Professor of Laryngology and Rhinology in the Chicago Post-Graduate Medical School. Vol. III. The Eye, Ear, Nose and Throat. Edited by Casey A. Wood, Albert H. Andrews and T. Melville Hardie. December, 1901. Pp. 346. Chicago: The Year Book Publishers. [Price, $1 50.]

Chemical Pathology in its Relation to Practical Medicine. By C. A. Herter, M. D., Professor of Pathological Chemistry in the University and Bellevue Medical College, New York, etc. In one 12mo volume of 454 pages. Philadelphia and New York: Lea Brothers & Co. 1902. [Cloth, $1.75 net.]

International Clinics. A Quarterly of Clinical Lectures and Especially Prepared Articles on Medicine, Neurology, Surgery, Therapeutics, Obstetrics, Pediatrics, Pathology, Dermatology, Diseases of the Eye, Ear, Nose and Throat, etc. Edited by Henry W. Cattell, A. M., M. D., of Philadelphia, with the collaboration of John B. Murphy, M. D., of Chicago; Alexander D. Blackader, M. D., of Montreal; H. C. Wood, M. D., of Philadelphia; T. M. Rotch, M. D. of Boston; E, Landolt, M. D., of Paris; Thomas G. Morton, M. D., and Charles H. Reed, M. D., of Philadelphia; J. W. Ballantyne, M. D., of Edinburgh, and John Harold, M. D.. of London Volume IV. Eleventh series. Philadelphia: J. B. Lippincott Co. 1902. [Price, cloth, $2.00 each; half-leather, $2.25 each ]

